# Factors associated with prehospital delay in acute myocardial infarction in Maldives

**DOI:** 10.1186/s12245-023-00503-2

**Published:** 2023-05-01

**Authors:** Madheeh Mohamed Hussain, Kamarul Aryffin Baharuddin, Mohd Hashairi Fauzi, Mimi Azliha Abu Bakar, Ahmed Ziyan, Aminath Zeyba Ahmed, Mohamed Sunil

**Affiliations:** 1 Department of Emergency Medicine, School of Medical Sciences, Universiti Sains Malaysia, Health Campus, 16150 Kubang Kerian, Kelantan, Malaysia; 2 Trauma and Emergency Department, Indira Gandhi Memorial Hospital, Malé, Republic of Maldives; 3 Hospital Universiti Sains Malaysia, Kubang Kerian, 16150 Kelantan, Malaysia; 4 National Cardiac Centre, Indira Gandhi Memorial Hospital, Malé, Republic of Maldives

**Keywords:** Prehospital delay, Myocardial infarction, Emergency medicine, Maldives

## Abstract

**Background:**

Acute myocardial infarction (AMI) is the top cause of death in Maldives. Our study aims to determine the prehospital delay and its associated factors in AMI patients in Maldives.

**Methods:**

A cross-sectional study was conducted with 127 patients, divided into early (≤ 6 h) and delayed (> 6 h) presenters to the hospital. The data collection for the study was carried out by interviewing AMI patients, focusing on their socio-demographic characteristics, coronary artery disease risk factors, clinical symptoms, situational factors, and behavioral and cognitive responses to symptoms.

**Results:**

The median onset-to-door time was 230 (IQR 420) minutes. The mean age of AMI patients was 50.9 (SD ± 12.9) years old, and 39.4% of them had delayed presentation to the hospital. Smokers (adj OR = 0.3; 95% CI: 0.1, 0.9; *P* = 0.047) and those with previous episodes of chest pain or AMI (adj OR = 0.2; 95% CI: 0.03, 0.91; *P* = 0.038) were significant factors for early presentation to the hospital, while denial of symptoms (adj OR = 29.3; 95% CI: 1.6, 547.2;* P* = 0.024) and lack of knowledge (adj OR = 7.2; 95% CI: 1.77, 29.43; *P* = 0.006) led to a delayed decision to seek treatment. Situational factors such as onset at the workplace (adj OR = 5.8; 95% CI: 1.24, 26.83; *P* = 0.025) had lower odds of delay, whereas referral cases (adj OR = 7.7; 95% CI: 1.9, 30.94; *P* = 0.004) and use of sea ambulance (adj OR = 11.1; 95% CI: 2.8, 43.8; *P* = 0.001) were prone to delay in presentation to the hospital.

**Conclusion:**

Sea ambulance, referral cases, lack of knowledge, and denial of symptoms are significant factors associated with prehospital delay among patients with AMI. Public awareness about the benefits of early presentation and improvement of the means of transportation between islands is suggested to improve emergency cardiac care in the country.

**Supplementary Information:**

The online version contains supplementary material available at 10.1186/s12245-023-00503-2.

## Introduction

The Republic of Maldives is a tiny nation in the South Asian region, located in the southwest of India and Sri Lanka in the Indian Ocean, with an area of 298 square kilometers. It consists of 1192 beautiful small islands grouped into 26 naturally formed atolls. With a population of an estimated 515,122 people [1], it has remarkable achievements in the health sector, notably eradicating malaria, measles, rubella, and controlling polio, neonatal tetanus, whooping cough, and diphtheria to an extent of nonexistence, while leprosy and filaria are reaching elimination targets [2].

However, non-communicable diseases (NCD) have been the leading cause of death in the Maldives for the past few decades. They have been topping the 20 leading causes of death for all ages, accounting for 79–84% of all deaths from year 2014 to 2016 [3, 4]. Coronary artery disease (CAD) has been the most frequent cause of death, accounting for 36% of all death [3].

The outcomes of CAD, particularly acute myocardial infarction (AMI), depends on the extent of myocardial salvage through early reperfusion therapy [5]. Therefore, any strategies to improve the time to reperfusion therapy, especially in the prehospital setting, are crucial [6]. Many studies have shown that the benefits of reperfusion therapy is largest during early presentation and is significantly reduced over time [7], with mortality and morbidity increasing considerably after 6 h of presentation [8, 9].

Recent studies have proven that prehospital delay is the major contributor to delayed treatment in South Asian countries such as India [10, 11], Pakistan [12, 13], Bangladesh [14], and Sri Lanka [15]. However, there is little or no information regarding the prehospital delay in the Maldives. A greater understanding of the contributing factors is necessary to improve this. With its unique geographic distribution in scattered small islands, Maldives faces a more significant challenge in the prehospital care system. It recently started developing a nationwide prehospital system with ambulances to all inhabited islands’ healthcare facilities, which was completed on 12 October 2019. The main tertiary hospital, Indira Gandhi Memorial Hospital (IGMH), started its prehospital care with the announcement of PRAMEDICs on 15 November 2020. However, transportation from peripheral hospitals to the capital city is still challenging. The sea-ambulance system is still functioning poorly and does not add much to prehospital care. Air transfer is still limited to chartered flights, and helicopter-based medical evacuations are rarely used. Therefore, scheduled commercial flight transfers remain among the most commonly used patient transfers from peripheral islands to the capital city.

Therefore, this study aims to determine the factors associated with prehospital delay in AMI patients by identifying patients’ socio-demographic data, clinical symptoms, first medical contact (FMC), behavioral and cognitive response to symptoms of AMI, means of transportation, situational factors, and onset-to-door time. Identification of these factors also helps to raise awareness and develop appropriate patient-targeted educational interventions and subsequently improve the quality of service of the healthcare system in the Maldives.

## Methods

### Study design

This was a cross-sectional study conducted from August 2018 to February 2020. Patients presented to the Trauma and Emergency Department (TED) at IGMH with a final diagnosis of AMI were enrolled. IGMH is the only tertiary government hospital with over 400 beds that manages approximately 150 patients per annum with AMI.

### Inclusion and exclusion criteria

All patients aged 18 years and above who developed symptoms of AMI outside the hospital, had a known time of onset of symptoms, and were diagnosed with AMI were enrolled in the study. The criteria to diagnose AMI are detection of the rise and/or fall in cardiac troponins, with at least one value above the 99th percentile of the URL, accompanied by at least one of the following [16]:

i. Clinical history consistent with chest pain of ischemic origin of > 30 min
ii. ECG changes in ischemia/infarction and/or the development of pathological Q waves
iii. Imaging evidence of new loss of viable myocardium or new regional wall motion abnormality
iv. Identification of an intracoronary (IC) thrombus by angiography or autopsy

Unconsented patients and patients who were unconscious at presentation were excluded from the study.

### Data collection

The data collection for the study was carried out by interviewing the patients who had been diagnosed with AMI at TED, IGMH. The interview took place once the patient had been stabilized and pain-free without interfering with the standard of care. The data were collected by the medical officer using the standard collection datasheet ( Additional file 1: Appendix). The patient’s relatives and family members were also interviewed for data verification. Arrival time was taken from the triage registry of TED, and the complete data collection sheet was counterchecked and signed by the field supervisor within 7 days of the patient’s arrival at the hospital. The data collection focused on the following:

i) Socio-demographic and CAD risk factor data: age, gender, nationality, educational level, hypertension, diabetes mellitus, hyperlipidemia (HPL), family history of CAD and smoking.
ii) Clinical symptoms: Character of chest pain (typical/atypical), severity of chest pain using a numerical rating scale, if patient attributed symptoms to heart, and previous episodes of similar pain.
iii) Behavioral and cognitive response to symptoms: Patients attempted any symptomatic relief measures, patients’ reason to decide to seek treatment, and reason for the delay of a decision to seek treatment.
iv) Situational factors: First medical contact (FMC) with a general practitioner (GP), health center/clinic, emergency department (ED), or pharmacist/over-the-counter medication. Place and time of symptom onset, being alone during symptom onset, referral and arrival time to TED, and means of transportation used—sea/land ambulance, air-transfer, taxi, self-drive and sent by another person by using personal vehicle, were evaluated in this category.

The onset-to-door time was calculated from the data collected and defined as the time interval from the onset of symptoms to the first documented time of arrival at TED. The time was calculated in minutes, and those presented after 6 h were categorized as delayed presenters, while those presented early were categorized as early presenters. The 6-h cut-off was chosen because the benefit of thrombolysis was most effective within the first hour and had a sharp decline after 6 h of the onset of symptoms [7, 17, 18]. Symptom-to-door time was calculated as the difference between arrival time to TED and time of onset of symptoms, and it is described as the median and interquartile range (IQR). Continuous data are expressed as the mean and standard deviation (SD) or median and IQR, while categorical data are expressed as numbers and percentages.

### Statistical analysis

IBM SPSS for Windows, version 24.0 (SPSS Inc. Chicago, IL, USA), was used to analyze the data. Factors associated with early (≤ 6 h) and delayed (> 6 h) arrival to the hospital were analyzed by simple logistic regression, and those variables with *p* value < 0.25 were proceeded to multiple logistic regression analysis. All variables that were analyzed with the adjusted odds ratio (adj OR) and 95% confidence interval (CI) in this category were reported, and a *p* value < 0.05 was considered significant.

### Ethical consideration

This study received ethical board approval from the Human Research Ethics Committee of Universiti Sains Malaysia (USM/JEPeM/18060285). The study protocol conformed to the ethical guidelines of the 1975 Declaration of Helsinki as reflected in prior approval by the human research committees. Permission to use the data was obtained from the patients by written consent.

## Results

The median onset-to-door time was 230 (IQR 420) minutes. From a total of 127 patients, 77 patients (60.6%) arrived early (˂ 6 h), while 50 patients (39.4%) had delayed (˃6 h) arrival at the hospital.

The mean age of AMI patients was 50.9 (SD ± 12.9) years old, ranging from 22 to 92 years old. The majority of the patients were male, and only 11 (8.7%) were female. Almost half of the patients had a primary schooling level of education, followed by secondary schooling, no schooling, and tertiary level of education.

The majority of the patients were smokers. CAD risk factors such as HPL, family history of CAD, diabetes mellitus, and hypertension were found in less than 34% of patients. Table 1 presents the demographic characteristics of the patients with AMI in the Maldives.

**Table 1:** Demographic characteristics of AMI patients in Maldives

Variables	*N* (%)
**Age** (years)^a^	50.9 (12.9)
**Sex**	
*Male*	116 (91.3)
*Female*	11 (8.7)
**Education level**	
*No schooling*	20 (15.7)
*Primary school*	58 (45.7)
*Secondary school*	30 (23.6)
*Tertiary education*	19 (15.0)
**CAD risk factors**	
*Hypertension*	
*Yes*	24 (18.9)
*No*	103 (81.1)
*Diabetes mellitus*	
*Yes*	27 (21.3)
*No*	100 (78.7)
*Family history of CAD*	
*Yes*	31 (24.4)
*No*	96 (75.6)
*HPL*	
*Yes*	34 (26.8)
*No*	93 (73.2)
*Smoking*	
*Yes*	80 (63.0)
*No*	47 (37.0)
*Other*	
*Yes*	9 (7.1)
*No*	118 (92.9)
**Nationality**	
*Maldivian*	101 (79.5)
*Foreigner*	26 (20.4)

ªMean (SD)

Analysis by the simple logistic regression in Table 2 identified several factors related to delayed presentation, including no history of chest pain/CAD, lack of knowledge about the importance of early treatment, referrals, and use of sea ambulance as means of transport.

**Table 2:** Factors associated with prehospital delay among AMI patients by simple logistic regression (*n* = 127)

Variables	Crude B	Crude OR (95% CI)	Wald	*p* value
**Age** (years)^c^	-0.03	0.97 (0.95, 1.00)	3.50	0.061
**Gender**				
*Male*		1		
*Female*	− 0.27	0.76 (0.22, 2.64)	0.19	0.666
**Education level**				
*No schooling*		1		
*Primary school*	0.09	1.09 (0.39, 3.09)	0.03	0.870
*Secondary school*	− 0.27	0.76 (0.24, 2.40)	0.22	0.642
*Tertiary education*	0.37	1.44 (0.39, 5.39)	0.30	0.584
**CAD risk factors**				
*Hypertension*				
*Yes*		1		
*No*	− 0.32	0.73 (0.29, 1.85)	0.45	0.503
*Diabetes mellitus*				
*Yes*		1		
*No*	− 0.13	0.88 (0.37, 2.12)	0.08	0.780
*Family history of CAD*				
*Yes*		1		
*No*	0.14	1.15 (0.51, 2.64)	0.11	0.737
*Hyperlipidemia*				
*Yes*		1		
*No*	0.27	1.31 (0.59, 2.90)	0.44	0.508
*Smoking*				
*Yes*		1		
*No*	− 0.49	0.61 (0.29, 1.28)	1.72	0.190
**Nationality**				
*Maldives*		1		
*Foreigner*	0.95	2.75	3.48	0.062
**Clinical characteristic**				
Previous episode of chest pain/CAD:				
*Yes*		1		
*No*	− 0.76	0.47 (0.21, 1.04)	3.51	0.061
Character of chest pain				
*Typical*		1		
*Atypical*	0.09	1.10 (0.37, 3.23)	0.03	0.870
Severity of chest pain				
*Mild (1–3)*		1		
*Moderate (4–6)*	0.93	2.54 (0.32, 20.38)	0.78	0.379
*Severe (7–10)*	0.24	1.27 (0.17, 9.45)	0.06	0.815
**Behavioral and Cognitive response:**				
Patients attempt at symptom relief measures:				
Waiting to resolve symptoms:				
*Yes*		1		
*No*	0.13	1.14 (0.56, 2.33)	0.12	0.727
Self/over-the-counter medication				
*Yes*		1		
*No*	0.42	1.53 (0.62, 3.79)	0.83	0.361
Denial of symptoms				
*Yes*		1		
*No*	1.89	6.61 (0.72, 60.95)	2.78	0.096
Patients reason to seek treatment:				
Attribute symptoms to heart				
*Yes*		1		
*No*	− 0.92	0.40 (0.18, 0.87)	5.37	0.020
Reason for delayed decision making:				
Attempting symptom relief				
*Yes*		1		
*No*	0.39	1.48 (0.65, 3.39)	0.87	0.351
Fear of troubling others				
*Yes*		1		
*No*	− 0.27	0.77 (0.07, 8.67)	0.05	0.829
Lack of knowledge				
*Yes*		1		
*No*	1.14	3.13 (1.20, 8.17)	5.41	0.020
**Situational factors:**				
Location of symptoms onset				
*Home*		1		
*Workplace*	1.07	2.93 (1.06, 8.09)	4.29	0.038
*Public place*	0.79	2.20 (0.77, 6.26)	2.17	0.141
*Others*	− 0.82	0.44 (0.04, 5.05)	0.44	0.509
First medical contact (FMC)				
*GP*		1		
*Health clinic*	− 1.12	0.33 (0.07, 1.58)	1.94	0.164
*Emergency department*	0.43	1.54 (0.34, 6.97)	0.31	0.576
Referred patient				
*Yes*		1		
*No*	2.33	10.31 (3.93, 27.09)	22.42	< 0.001
Means of transportation				
*Ambulance*		1		
*Taxi*	2.30	9.92 (2.79, 35.30)	12.56	< 0.001
*Send by another person*	1.20	3.31 (0.63, 17.36)	2.00	0.157
*Self-drive*	1.01	2.76 (0.51, 15.03)	1.37	0.241
Air transfer				
*Yes*		1		
*No*	3.47	32.14 (4.08, 253.11)	10.86	0.001
Ambulance				
*Yes*		1		
*No*	− 1.39	0.25 (0.10, 0.64)	8.44	0.004
Land ambulance				
*Yes*		1		
*No*	1.79	5.99 (2.40, 14.95)	14.68	< 0.001
Sea ambulance				
*Yes*		1		
*No*	1.90	6.70 (2.82, 15.92)	18.56	< 0.001

Multiple logistic regression identified the following factors that significantly delayed presentation to the hospital: lack of knowledge about the importance of early presentation (adj. OR 7.2, *p* = 0.006), sea ambulance (adj. OR 11.1, *p* = 0.001), referred case (adj. OR 7.7, *p* = 0.004), and denial of the symptoms (adj. OR 29.3, *p* = 0.024) as shown in Table 3. Non-smokers were 3.3 times more likely to experience delayed arrival at the hospital than smokers. On the other hand, those who had a previous episode of chest pain had a five-fold odds ratio (*p* = 0.038) of early arrival at the hospital compared to those who had no experience of chest pain or CAD. Symptom onset at the workplace led to 5.8 times earlier arrival to the hospital, than those who had experienced the first symptoms at home.

**Table 3:** Factors associated with prehospital delay among AMI patients (*n* = 127) (multiple logistic regression)

Variables	Adjusted B	Adjusted OR (95% CI)	Wald	*P* value
Previous episode of chest pain/CAD				
*Yes*		1		
*No*	− 1.75	0.2 (0.03, 0.91)	4.3	0.038
Lack of knowledge				
Yes		1		
No	2.0	7.2 (1.77, 29.43)	7.6	0.006
Location of symptoms onset				
*Home*		1		
*Workplace*	1.8	5.8 (1.24, 26.83)	5.0	0.025
*Public place*	0.5	1.7 (0.43, 6.87)	0.6	0.446
With referral				
*Yes*		1		
*No*	2.04	7.7 (1.9, 30.94)	8.2	0.004
Sea ambulance				
Yes		1		
No	2.4	11.1 (2.8, 43.8)	11.8	0.001
Denial of symptoms				
Yes		1		
No	3.4	29.3 (1.6, 547.2)	5.1	0.024
*Smoking*				
*Yes*		1		
*No*	− 1.2	0.3 (0.1, 0.9)	3.9	0.047

Multicollinearity and interaction terms were checked and not found. Hosmer–Lemeshow test (*P* = 0.215). The classification table was 70.3%, and the predicted probability with the ROC curve was 80.4% (95% CI: 72.1%, 88.7%, *P* value < 0.001)

## Discussion

Analysis of the demographic factors in Table 1 revealed that male gender and old age had a predominance over other characteristics of AMI patients. More than 90% of the patients were male, which is significantly higher than the worldwide prevalence and neighboring countries. India has a male dominance, ranging from 78.6 to 81% [11, 19], while Sri Lanka has 69.9% male patients [20]. The mean age of the Maldivian patients was 50.9 years old, which is slightly younger than that of Sri Lankan and Indian patients. The mean age in Sri Lanka ranges from 54 to 61 years old [20, 21], and in India, it ranges from 55 to 59 years old [19, 22].

It was found that the youngest patient in Maldives was a 22-year-old foreigner from Bangladesh, while the youngest Maldivian patient was a 30-year-old man. To date, no studies have explored the youngest age of AMIs among Maldivian patients, in comparison to Bangladesh, where the youngest documented patient was 17.5 years old [23]. Based on the experience of cardiologists at IGMH, the youngest patient was 21 years old prior to COVID-19 pandemic. The findings of our study are worrying for as it might suggest a rising incidence of AMI amongst young patients.

Table 1 also shows that more than 60% of the CAD patients were smokers. The GREEC observational study found a positive association of smoking with CAD [24].

Data from the WHO-South Asia region have shown that the mean age of initiation of smoking among Maldivians is 17.8 years old [25]. Furthermore, almost 40% of Maldivians are either tobacco smokers or users. Maldives also have a far higher percentage of cardiovascular deaths attributable to tobacco in different age groups, with 23.9% compared to 15% and 16% for Sri Lanka and India, respectively [25]. This information could possibly explain the prevalence of CAD in young patients.

Data from neighboring countries suggested that smoking is not associated with prehospital delay [22, 26]. However, our study showed that smokers were presented to the hospital earlier than non-smokers. This finding is similar to a study from Japan where smoking was associated with early hospital presentation [27]. One of the explanations is that smokers tend to experience more intense pain than non-smokers [28].

Early arrival for AMI patients to the hospital is a pivotal point for therapy. The median onset-to-door time is 230 min. This finding is comparable with neighboring countries such as Sri Lanka and India, where the ranges of time are 190–420 min and 250–420 min, respectively [15, 22]. Some of the main reasons for the delay in Sri Lanka are the absence of a centralized referral system, problems with transportation, and lack of ambulance services [21]. In India, factors associated with delayed presentation are elderly patients, female gender, lack of awareness, problems with transportation, and money-related issues [11, 19, 22]. In this study, the number of patients who arrived within 6 h was high (60.6%) compared to that in India (50.3%) [29] and that in Bangladesh (39.5%) [30]. There was a significant delay in presentation for patients transported by airplane from Atolls, which sometimes took more than 1 h. The longest onset-to-door time was 1500 min. This is mostly because of waiting for scheduled commercial flights with fixed departure times without special arrangements, even for emergency cases. However, even with the unique geographical distributions and problems with transportation, 60% of the patients who arrived early in the hospital can be considered a good result.

The onset-to-door time in developed countries is much less than that in South Asian countries. For comparison, the UK, South Korea, and Australia have door-to-needle times of 120, 180, and 132 min, respectively [31, 32]. Well-developed prehospital care is the contributing factor to the results and has also shortened the time for countries such as America [33], Belgium [34], and Sweden [35].

Table 3 shows that patients with previous episodes of chest pain had 5 times the odds of presenting early to the hospital compared to those who did not have previous experience. This is comparable with the findings from India [29]. Knowledge and previous experience of the patient lead to awareness and fear of the consequences of AMI.

Generally, patients’ behavioral and cognitive responses toward the symptoms of AMI have been associated with prehospital delay. Some studies have related it to prehospital delay [14], while others have found that it minimizes the delay [36, 37]. Our study analyzed seven variables under these categories and found that two variables were related to prehospital delay. Those patients who denied symptoms and carried on their daily activities had 29.3 times the odds of being delayed in presenting to the hospital, which is consistent with findings from previous studies from Germany, China, and Malaysia [38, 39]. Half of the China PEACE study cohort perceived the symptom as not bad enough for emergency care, and it would go over time [40]. One recent study found that patients with chest pain characteristics such as recurrent episodes, short duration (less than 5 min), and aggravation by breathing, movement, palpitation, or exercise have a high likelihood of becoming late presenters [41].

Lack of knowledge about the benefits of early presentation and treatment was one of the reasons for the delay in seeking medical treatment. Those with a lack of knowledge had 7.2 times the chance of being delayed in presenting to the hospital. This is supported by our regional statistics, which relate to the low educational levels and awareness about the early presentation of AMI [11, 12]. This result is comparable with India [22], Pakistan [12], Australia, and New Zealand [11].

For the situational factors, the onset of symptoms at the workplace had less odds of delay by 5.8 times compared to those at home. A likely explanation for this is the better understanding of symptoms of AMI among educated co-workers and the caring nature of their peers. However, patients who were being referred from other hospitals had a significant delay with a 7.7 times chance of delay in the presentation. This is contributed by the difficulties in obtaining urgent transportation due to waiting for the scheduled commercial flights. The referral delay is also a well-known factor in the region, as in India, where outpatient clinic referrals have a significant delay [42]. Those who are transferred by sea ambulance have 11.1 times more chances of delay in the presentation to the hospital. Delays in transportation are one of the system delays in the healthcare services of Maldives. A proper prehospital care system, such as a well-functioning EMS system and a medical air-evacuation system by means of helicopters, may shorten prehospital delays, as is well known in developed countries such as Europe. It is also considered an unmet need for emergency care and must be improved to reduce prehospital death in AMI [43].

## Limitations

Our study has a relatively small number of cases from a single center, where there are two centers capable of PCI in Maldives. Nevertheless, IGMH is the only government center and caters to most of the population in Maldives.

With regard to the onset-to-door time, it is difficult to exclude the influence of recall bias on the time of onset of symptoms. Many studies have reported the time as self-reported, as it is difficult to exactly identify the time of onset [44, 45]. Therefore, to minimize this limitation, only fully conscious patients were enrolled in the study. Another limitation is that our study did not examine the outcome of AMI patients with regard to the factors associated with prehospital delay.

## Conclusion

Prehospital delays among AMI patients in Maldives are due to delays in sea ambulance services and referral processes from other hospitals. Lack of knowledge and denial of the symptoms also contribute to the delay. Previous experience of AMI and onset of symptoms in the workplace lead to earlier presentation to the hospital. Public awareness about the benefits of early presentation and improvement of the means of transportation between islands with a proper EMS system and an air-transfer system are crucial to improve the time of arrival and eventually prevent prehospital death.

## Supplementary Information

**Additional file 1.**

## Data Availability

The datasets generated during and/or analyzed during the current study are available from the corresponding author on reasonable request.
