# Supplementary material for: Factors associated with prehospital delay in acute myocardial infarction in Maldives
Source: Int J Emerg Med. 2023 May 1;16:31. doi: 10.1186/s12245-023-00503-2 (PMC10149151; doi:10.1186/s12245-023-00503-2)
Supplement: Supplementary file 1 — Additional file 1. [file 12245_2023_503_MOESM1_ESM.docx]

**Appendix I**

| 1. Patient’s Hospital No |  | |
| --- | --- | --- |
| 1. Age/Nationality |  | |
| 1. Gender | Male Female | |
| 1. Education Level | Primary school Secondary school  Tertiary Education | |
| 1. Comorbidities and risk factors | Hypertension DM  Family history of CAD Hyperlipidemia  Smoking Others : | |
| 1. *Time of symptoms onset | …………….. Hours Date: ……/……/…………. | |
| 1. Time of decision to seek treatment from the onset of symptoms? | ………………… minutes | |
| 1. Chest pain | Typical Atypical | |
| 1. Patient attributed symptoms to heart? | Yes No | |
| 1. Severity/pain score: | No pain (0) Mild (1-3) Moderate (4-6) Severe (7-10) | |
| 1. **Previous episodes? | Yes | No |
| 1. Patients attempt at symptom relief measures? | Waiting to resolve symptoms  Denial of the symptoms  (carrying out daily  work/job with denial of pain) | Self medication/Over-the-counter medication  Others…………. |
| 1. Patients reason to decide to seek treatment? | Progression of symptoms | Attribute symptoms to  cardiac cause |
| 1. Reason for delay of decision to seek treatment?   (If Patient arrive after 6 hours of Symptom onset) | 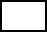Fear of consequences  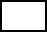Alone at onset of symptoms  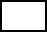Fear of ambbrassment should it turn out to be “ false alarm ”. | 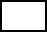Attempting symptom relief  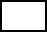Fear of troubling otheres  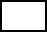Lack of knowladge about importance of early treatment. |
| 1. Place of onset of symptoms | Home | Workplace |
|  | Public places | Others: |
| 1. Patient alone during the onset of symptoms? | Yes | No |
| 1. First medical contact (FMC) | General practitioner | Health Centre/Clinic |
|  | Emergency Department | Self-bought medication/  Over counter |
|  | Others : | |
| 1. Referral | Yes No  Reffering center:  Date &Time. | |
| 1. Means of Transportation | Ambulance (land, air or sea) Taxi  Send by another person  using personal vehicle Self-drive | |
| 1. Time of arrival at TED | ……………….. Hours | |
| 1. Time of arrival at CCU | ……………….. Hours | |
| 1. Time of arrival at Cath lab | ……………….. Hours | |
| 1. Door to ECG time | ………………. minute | |
| 1. Door to needle time | ………………. minute | |
| 1. Door to balloon time | ………………. minute | |
| ** *24. Onset to door time | ………………. minute | |

* Time of symptom onset:

- Time when the patient first notes ischemic symptoms lasting 10 minutes or longer.

-If the patient has intermittent symptoms, the time of most recent symptoms before arrival at the hospital.

- If symptoms are varying in quality or intensity then the time is taken when the symptoms are persistent/constant.

** Previous episode: history of similar symptoms or history of acute coronary syndrome (ACS)

*** Onset to Door time: time from symptom onset to arrival at the Trauma and Emergency Department, IGMH
